# Scrapie incidence and *PRNP* polymorphisms: rare small ruminant breeds of Sicily with TSE protecting genetic reservoirs

**DOI:** 10.1186/s12917-016-0766-9

**Published:** 2016-07-15

**Authors:** Maria Vitale, Sergio Migliore, Maria La Giglia, Placido Alberti, Vincenzo Di Marco Lo Presti, Jan P. M. Langeveld

**Affiliations:** Istituto Zooprofilattico Sperimentale of Sicily “A. Mirri”, Palermo, Italy; Department of Infection Biology, Central Veterinary Institute of Wageningen UR, Lelystad, The Netherlands

**Keywords:** Scrapie surveillance, Autochthonous breeds, *PRNP* polymorphism, Breeding programs, TSE

## Abstract

**Background:**

Transmissible spongiform encephalopathies (TSE) are fatal neurodegenerative diseases of several mammalian species, including humans. In Italy, the active surveillance through rapid tests on brain stem from small ruminants started in 2002 on randomly selected samples of healthy slaughtered animals. Sampling number was proportionally related to the regional small ruminant population. Of the twenty Italian regions, Sicily has the second largest population of small ruminants which is mainly constituted by crossbreed animals (>70 %). Sicily contains also three native sheep breeds *Pinzirita*, *Comisana* and *Valle del Belice.* Native goat breeds are *Girgentana*, *Messinese*, *Argentata dell’Etna, Maltese* and *Rossa Mediterranea.* The polymorphisms of prion protein gene (*PRNP)* may influence disease susceptibility and breeding programs for genetic TSE resistance are being applied in sheep. Protective alleles have been recently reported for goats also. These differ from those in sheep and may allow breeding programs in the near future.

In this paper the data of active surveillance for scrapie control in general population of small ruminants in Sicily are reported together with the analysis on the polymorphism of *PRNP* in a number of Sicilian autochthonous breeds. The evaluation of the frequency of protective alleles is fundamental for the implementation of a TSE resistance breeding program.

**Results:**

TSE surveillance in small ruminants in Sicily showed a of total fifty seven scrapie outbreaks from 1997 to 2014 involving mainly crossbreed animals. The *PRNP* polymorphism analysis in autochthonous breeds showed protective allele frequencies of 30–40 % ARR in sheep and 12–18 % K222 in three of the four goat breeds; these breeds are distributed over limited areas of the island.

**Conclusion:**

The study on *PRNP* polymorphisms in Sicilian small ruminant population showed higher frequency of the protective alleles compared to most other European breeds. Our results suggest that *PRNP* genetic variety in Sicilian sheep and goats can be a resource for TSE resistance breeding programmes while maintaining the conservation of endangered breeds and valorisation of their typical food products.

**Electronic supplementary material:**

The online version of this article (doi:10.1186/s12917-016-0766-9) contains supplementary material, which is available to authorized users.

## Background

Transmissible spongiform encephalopathies (TSE) are fatal neurodegenerative diseases known to affect several mammalian species, including humans and ruminants. TSEs in ruminants include scrapie in sheep and goats, bovine spongiform encephalopathy (BSE) in cows and chronic wasting disease (CWD) in cervids. Scrapie is not regarded as a human health risk, whereas BSE has been recognized as the cause of a new TSE in humans defined as variant Creutzfeldt-Jakob disease (vCJD) [1, 2].

The second largest Italian small ruminants population is present in Sicily with about 880 thousands sheep and 132 thousands goats reared in 11,416 farms [3]. A significant biodiversity is represented by three native dairy sheep breeds (*Comisana, Valle del Belice* and *Pinzirita*) and five native dairy goat breeds (*Girgentana, Messinese*, *Argentata dell’Etna, Maltese* and *Rossa Mediterranea* or *Derivata di Siria)*, in addition to other Italian breeds*.* These breeds have an important role in the production of high quality milk requested for typical dairy products.

Prion protein (PrP) is encoded by *PRNP* gene and is strongly conserved among mammals [4]. Mutations and polymorphisms of *PRNP* sequences may influence disease susceptibility through modified protein conformation. The PrP polymorphic forms are equally expressed, however in prion material the deposition of the polymorphic-variant PrP^Sc^ can differ between two allotypes in a heterozygous individual [5–7].

In various sheep breeds the level of risk for scrapie is associated with *PRNP* genotype [8] particularly at the three codons: 136 (alanine or valine; A, V), 154 (histidine or arginine; H, R), and 171 (glutamine, arginine or histidine; Q, R, H). *PRNP* haplotypes VRQ and ARQ are considered the most susceptible to classical scrapie, whereas the ARR allele has been linked to resistance [9, 10].

In goats the analysis on *PRNP* polymorphisms showed that some polymorphic variants can be protective for the disease but their prevalence in different breeds are still under investigation. The polymorphisms that might be related to TSE resistance are present at *PRNP* codon 222 with a lysine (K) instead of glutamine (Q) [11–16] and at codon 146 with a serine (S) or an aspartic acid (D) instead of asparagine (N) [17, 18]. Strong evidence by experimental challenges in goats, rodents had shown that with the K222 allele a high level of TSE-resistance can be reached even if present in heterozygous animals [19–22].

The active surveillance by rapid diagnosis in sheep and goats started in EU in 2002 on random sampled animals. Fallen and healthy slaughtered animals over 18 months of age were included for a total number of tests that has varied each year. In Italy from 2002 to 2014, 656 positive flocks were detected on a total 620,000 tested animals (Italian Reference Centre for Animal Encephalopathy, personal communication).

The overall results of TSE surveillance and *PRNP* polymorphisms studies in native small ruminant breeds of Sicily are reported in this paper to evaluate the biodiversity of *PRNP* gene related to TSE resistance in autochthonous breeds.

## Methods

### Rapid diagnosis test on regularly slaughtered animals

The data on TSE surveillance reported in the paper are referred to the routine activity on the slaughtered animals for TSE control under the regulation (EC) No 999/2001, laying down rules for the prevention, control and eradication of certain transmissible spongiform encephalopathies. Almost 102 thousand regularly slaughtered sheep and goats older than 18 months have been tested in Sicily from April 2002 to December of 2014 (Italian Reference Centre for Animal Encephalopathy, personal communication). The majority of tested animals were crossbreeds because pure breeds represent a very small percentage of the zootechnical patrimony in the island. Sampling was randomly performed each year at the abattoirs to reach the number required by the national surveillance plan. The number of tests was related to the regional population of sheep and goat. Rapid diagnosis test for TSE was performed through the years by either Western blot (The Prionics®-Check WESTERN), ELISA (TeSeE Purification and Detection Biorad), chemiluminescent Elisa (Enfer TSE ELISA –Abbott USA) or in more recent years by IDEXX EIA following manufacturer’s instructions.

### Sampling and molecular analysis for polymorphism studies in native breeds

Consent for the sampling was obtained from the animal’s owners and blood collection was performed according to the principles of Good Clinical Practice (VICH GL9 GCP, 2000).

Sheep: analysis were performed on a total of 1,295 individuals of *Valle del Belice*, 1,341 *Comisana* and 84 *Pinzirita* sheep from several flocks in different Sicilian provinces. Some flocks were analysed under personal request of veterinarians and breeders some according to the first Sicilian regional plan for genetic resistance selection.

Goat: analysis were performed on 504 pure breed animals, specifically 179 *Girgentana*, 102 *Rossa Mediterranea*, 80 *Argentata dell’Etna* and 143 *Maltese* enrolled for the specific collaboration of goat breeders.

DNA extraction from blood samples for *PRNP* polymorphism analysis was performed with E.Z.N.A Tissue-DNA-kit (Omega®) and Biosprint® 96 One-For-All Vet kit (Qiagen®) according to the manufacturer’s protocol.

Sheep genotyping was performed by reverse hybridization method using the Kit Ovine PrP Gene Test, from Nuclear Laser Medicine, following manufactures’ instructions and by allelic discriminations by real-time PCR with TaqMan probes in Applied Byosystem 7700. A couple of TaqMan probes (with FAM and VIC) for each SNP were used in single PCR reaction for the codon 136, 154 and 171 respectively, involved in disease susceptibility. In the homozygous animals only FAM or VIC signal can be detected while in the heterozygous both signals are present.

The sequencing on the amplified coding sequence of goat PrP gene was performed by Kit BigDye Terminator v3.1 Cycle Sequencing and ABI prism 3130 genetic analyzer (Applied Biosystem).

### Statistical analysis

Statistical analysis were performed using Chi-Square test (2x2 Contingency table). ARR allele was compared among the main two sheep breeds and K222 variant among the goat breeds with a significance level of P ≤ 0.05. Fisher exact test was used for *Maltese* goat that showed a low K222 frequency (0.3 %)

## Results

In a small ruminants population of 1,023,919 heads [3], 57 scrapie outbreaks had been detected up to the end of 2014; only one sheep and one goat were classified as atypical cases. Outbreaks involved 30 sheep flocks, 5 goat flocks and 22 mixed flocks of sheep and goats together which is a common farming situation in Sicily. During the active surveillance on a total of almost 102,000 tested animals, 50 scrapie outbreaks were detected with an incidence of 12.24 cases per 10,000 tested animals. The majority of scrapie positive sheep were crossbreeds of mixed genetic background (mostly Sicilian autochthonous plus *Sarda* sheep), while the autochthonous animals concerned one *Pinzirita*, one *Valle del Belice* and four *Comisana* outbreaks (Table 1). Genetic polymorphism analysis in sheep had shown that almost all TSE positive individuals were of ARQ/ARQ *PRNP* genotype which is the most frequent in classical scrapie cases in Italy; less than 2 % of positive animals had VRQ/ARQ and ARQ/AHQ genotypes. The analysis in Sicilian sheep breeds on a total of 1395 individuals of *Valle del Belice*, 1541 *Comisana* and 84 *Pinzirita* showed that the susceptible haplotype ARQ is present at almost 59, 48 and 59 % while the resistant ARR at 32.8, 40.4 and 33.3 % respectively (Table 2). We compared the ARR allele frequencies between the group of *Valle del Belice* and *Comisana* respectively using the Chi-Square test and the result was significant with a *p-value* of 0.010 and 0.023 in *Valle del Belice* and *Comisana* breeds respectively (Table 2). Statistical analysis in *Pinzirita* sheep was not carried out because the sample was not representative of the whole population, however *Pinzirita* genetic background is present in *Valle del Belice* which was derived from a crossing between *Pinzirita-Comisana* and *Sarda* sheep to increase milk production.

**Table 1:** Scrapie outbreaks reported in Sicily from 1997 to 2014^a^

year	# outbreaks	affected species	sheep breeds	goat breeds
1997	3	ovine-caprine	*Pinzirita* - crossbreed	*Argentata dell' Etna* - crossbreed
1998	1	ovine-caprine	*Comisana*	*Rossa Mediterranea*
1999	1	ovine	*Sarda*	
2000	2	ovine	*Bergamasca* - crossbreed	
2002	6	ovine-caprine	*Comisana* - crossbreed	*Rossa Mediterranea - Maltese* - crossbreed
2003	5	ovine-caprine	*Comisana - Pinzirita -Sarda* - crossbreed	crossbreed
2004	4	ovine-caprine	*Pinzirita* - crossbreed	crossbreed
2005	8	ovine-caprine	*Comisana* - crossbreed	*Rossa Mediterranea* - crossbreed
2006	7	ovine-caprine	*Valle del Belice* - crossbreed	*Rossa Mediterranea* - crossbreed
2007	7	ovine-caprine	*Comisana* - crossbreed	crossbreed
2008	1	ovine-caprine	crossbreed	crossbreed
2011	2	ovine-caprine	crossbreed	*Girgentana* - crossbreed
2012	6	ovine-caprine	*Sarda* - crossbreed	*Rossa Mediterranea* - crossbreed
2013	2	ovine	crossbreed	
2014	2	ovine	*Sarda* - crossbreed	

^a^ Scrapie outbreaks were detected through passive surveillance (symptomatic animals) from 1997 to 2000 and through active surveillance by rapid diagnosis on stem-brains from 2002 to the end of 2014

**Table 2:** Haplotype frequencies of PRNP in Sicilian autochthonous sheep breeds^a^

PRNP codons			Sheep breeds		
**136**	**154**	**171**	***Valle del Belice (%)***	***Comisana (%)***	***Pinzirita (%)***
A	R	Q	58.9	47.8	59.2
A	R	**R**	**32.8**	**40.4**	**33.3**
	***p-value***		*(0.010)*	*(0.023)*	
A	R	**H**	0.8	2.8	-
A	**H**	Q	5.3	1.7	3.3
**V**	R	Q	2.2	7.3	4.2

^a^ The analysis was performed on a total of 1395 individuals of *Valle del Belice*, 1541 *Comisana* and 84 *Pinzirita* sheep. The haplotypes are presented in decreasing frequency; in bold are the polymorphic variant amino acid versus wild type (ARQ). For ARR haplotype we performed statistical analysis using Chi Square test with a significant level of p ≤ 0.05. In brackets the *p-value* results. Statistical analysis in *Pinzirita* was not performed

The application of breeding programs in goats could also aid the ongoing EU efforts to full eradicate scrapie. The investigation into the relationships between caprine PRNP haplotypes and resistance to scrapie in goat breeds is important for the design of an effective breeding program. Our study on autochthonous goat breeds showed an 18.7 % frequency of the protective variant K222 in *Girgentana* [23], 12 % in *Rossa Mediterranea*, 16 % in *Argentata dell’Etna* and 0.3 % in *Maltese* (Table 3). Statistical analysis among goat breeds for the presence of K222 variant was performed with Chi-Square test considering each breed versus all goat samples. In *Maltese* goat which has a low K222 frequency we used Fisher exact test. The results were significant in *Girgentana* and *Maltese* breeds with a *p-value* of 0.047 and 0.002 respectively. In contrast, in *Rossa Mediterranea* and *Argentata dell’Etna* a *p-values* of 0.933 and 0.306 were obtained respectively (Table 3).

**Table 3:** Allelic frequencies in Sicilian autochthonous goats breeds^a^

Allele	Girgentana (%)	*Rossa Mediterranea (%)*	*Argentata dell’Etna (%)*	*Maltese (%)*
37V	0.3	1.5	-	24.1
125V	-	-	1.3	-
137I	-	-	2.5	0.3
143R	3.8	2.5	-	0.7
151H	-	-	2.5	-
154H	7.6	7.4	13.8	5.6
168Q	18.4	3.4	2.5	-
222K *p-value*	18.7 *(0.047)*	12.7 *(0.933)*	16.3 *(0.306)*	0.3 *(0.002)*
240P	57.3	51.0	51.3	52.1

^a ^The analysis was performed on 179 *Girgentana*, 102 *Rossa Mediterranea*, 80 *Argentata dell’Etna* and 143 *Maltese*. For the presence of K222 variant we used Chi-square test with a significant level of p ≤ 0.05 in *Girgentana*, *Rossa Mediterranea* and *Argentata dell’Etna*. In *Maltese* we used Fisher exact test with a significance level of P ≤ 0.05

## Discussion

The results of passive and active surveillance had shown how 57 scrapie outbreaks have occurred in the period from 1997 to 2014 in Sicily. The disease probably became endemic in the island when an infected vaccine against *Mycoplasma agalactiae* was used in small ruminant flocks [24]. Scrapie is not regarded as a human health risk, but to reduce the risk of scrapie or BSE in sheep the EU decided to implement selection against susceptible *PRNP* alleles (Commission Regulation EC 999/2001 and Commission Decisions 2002/1003/EC3 and 2003/100/EC4). Since then, several member states established breeding programs to enrich for resistant alleles. In Sicily a regional public plan for genetic selection in sheep started in 2005 for pure breed animals and was extended to cross breed flocks with more than 200 animals in 2013. In addition, genotyping of several flocks had started in 2005 on voluntary basis particularly to manage scrapie outbreaks. A previous study on limited number of pure breed sheep flocks (*Valle del Belice* and *Comisana*) and cross breed animals from five outbreaks showed already a good level of ARR haplotype frequency [25]. The results of a more extended analysis on reproductive males and females and young animals from pure breed sheep flocks in different Sicilian provinces confirmed the high frequency of the resistant haplotypes (Table 2). It is widely recognized that stamping out in sheep is not an efficient strategy for TSEs eradication probably because the pathogenic prion protein is highly persisting in the environment and naïve animals can acquire the infection from the soil [26]. In contrast the selective breeding program is a good way to manage scrapie outbreaks and the best alternative to stamping out of animals which represents a great economic loss for the farmers and a serious threat of extinction for the endangered breeds [27, 28]. The application of breeding programs in goats could also aid the ongoing EU efforts to reduce the chance of scrapie in sheep populations since, goats may represent a possible scrapie reservoir for sheep. This would theoretically also apply if goats were positive for the zoonotic BSE strain [29–31]. In Europe, very few cases of goat scrapie had been recorded prior to 2002 but in Sicily the first clinical case in goat was reported in a mixed flock in 1997 and 27 outbreaks have been diagnosed with 9 including pure breed animals. Any breeding program should consider the endangered status of each goat population to preserve the genetic variability and the biodiversity also when dealing with disease control. For these reasons, the investigation into the relationships between caprine *PRNP* haplotypes and resistance to scrapie in goat breeds is important [11–17, 31]. With the K222 allele a high level of TSE-resistance can be reached even if present in heterozygous animals [19–22]. Except for *Maltese,* all other Sicilian goats presented K222 variant in frequency higher than most commercial breeds although more analysis are necessary for *Argentata dell’Etna* and *Rossa Mediterranea.*

Our results suggest that a breeding program to select for ARR haplotype in sheep and K222 resistant allele in goat is feasible in the autochthonous breeds of Sicily.

Small ruminants are bred worldwide, particularly in difficult and marginal geographical territories (mountains or dry areas) to which they are well adapted. The native breeds are usually confined but important in low-income rural areas even in developed countries. The focus on increasing biosecurity and quality of local animal production can stimulate the breeding and protection of autochthonous animals through a revival of their economic value as suggested by FAO [32].

## Conclusions

The high prevalence of the two TSE resistance related ARR haplotype and K222 allele present in Sicilian sheep and goat breeds respectively, shows how the genetic biodiversity could serve as a resource for the selection of resistance to disease, in our case for TSE eradication. It is very probable that enhancing the frequencies of resistant polymorphisms could be sufficient to reach a sufficiently high level of heterozygosis with the advantage to maintain both biodiversity and resistance. Genetic selection for resistance could assure the full eradication of scrapie in small ruminants. These results may increase interest in the breeding of Sicilian autochthonous breeds preserving the biodiversity on the island. Additionally the promotion of typical dairy and meat products with high quality and biosecurity from autochthonous animals might represent a driving force for the economy in rural areas.

## Abbreviations

amino acid code, A, alanine; V, valine; R, arginine; Q, glutamine; H, histidine; K, lysine; S, serine; D, aspartic acid; N, asparagine; BSE, bovine spongiform encephalopathy; CWD, chronic wasting disease; DNA, deoxyribonucleic acid; ELISA, enzyme linked immunosorbent assay; EU, European Union; FAO, Food and Agriculture Organization; PCR, polymerase chain reaction; *PRNP*, prion protein gene; PrP, prion protein; PrPs, scrapie prion protein; TSE, transmissible spongiform encephalopathy; vCJD, vCreutzfeldt-Jakob disease

## Additional files

Additional file 1: Total number of analysed animals for each Sicilian sheep breed with allele frequencies and percentages. (XLS 56 kb) Additional file 2: Total number of analysed animals for each Sicilian goat breed with single polymorphism frequency and percentage. (XLS 56 kb)
